# Intramuscular Fat Influences Neuromuscular Activation of the Gluteus Medius in Older Adults

**DOI:** 10.3389/fphys.2020.614415

**Published:** 2020-12-10

**Authors:** Marcel B. Lanza, Alice S. Ryan, Vicki Gray, William J. Perez, Odessa Addison

**Affiliations:** ^1^Department of Physical Therapy and Rehabilitation Science, University of Maryland School of Medicine, Baltimore, MD, United States; ^2^Division of Gerontology and Geriatric Medicine, Department of Medicine, University of Maryland School of Medicine, Baltimore, MD, United States; ^3^Baltimore Geriatric Research, Education, and Clinical Center (GRECC), The Veterans Affairs Maryland Health Care System, Baltimore, MD, United States

**Keywords:** intramuscular fat, muscle activation, isometric contraction, older adults, aging, surface electromyography

## Abstract

The amount of tissue between the muscle and surface electromyography (sEMG) electrode influences the sEMG signals. Increased intramuscular adipose tissue (IMAT) of the hip abductor muscles negatively impacts balance in older individuals, but it is unknown if this is related to the ability to activate the muscles. The aim of this preliminary study was to investigate the influence of gluteus medius (GM) IMAT on sEMG amplitude during maximal voluntary isometric contractions (MVIC) of the hip abductors in older adults. We recruited 12 healthy community-dwelling older adults that underwent a spiral computerized tomography scan. High density lean (HDL), IMAT, and subcutaneous adipose tissue (SUBFAT) cross-sectional area of the GM were assessed. sEMG signal from the GM was recorded while participants performed an MVIC of the hip abductors. There was a negative correlation between GM activation and IMAT (*r* = −0.58, *P* = 0.046), and also SUB_FAT_ (*r* = −0.78, *P* = 0.002) and a positive correlation with HDL (*r* = 0.73, *P* = 0.006). When controlling for SUB_FAT_, the partial correlations demonstrated a consistent negative correlation between GM activation and IMAT (*r* = −0.60, *P* = 0.050) but no relationship with HDL. The current results are important for helping to interpret the results from sEMG by accounting for IMAT. In conclusion, the neuromuscular activation of the GM may be reduced by the quantity of IMAT.

## Introduction

Surface electromyography (sEMG) is widely used to assess neuromuscular activation across multiple populations, including healthy individuals and those with neurological disorders (Vandervoort, 2002; Hogrel, 2005). Multiple intrinsic factors may influence recording signals from sEMG, such as fiber composition, blood flow, and the amount of tissue between the muscle and EMG electrode (De Luca, 1997; Lanza et al., 2018). The amount of subcutaneous fat between the muscle and the electrode is well known to influence sEMG amplitude (Petrofsky, 2008; Lanza et al., 2018). It is theorized that inhomogeneity in the muscle, such as increased amounts of adipose tissue inside the muscle, may also impact sEMG amplitude.

Intramuscular adipose tissue (IMAT), or the amount of adipose tissue within the fascia of a muscle, increases with age and inactivity (Addison et al., 2014a; Correa-de-Araujo et al., 2020), and is an important factor influencing muscle function and quality (Addison et al., 2014a; Biltz et al., 2020). Furthermore, increased IMAT is associated with decreases in strength (Reid et al., 2012) and mobility (Bamman et al., 2000; Newman et al., 2006) and reduces the ability to recover balance in older adults (Addison et al., 2014b). For example, increased IMAT of the hip abductor muscles negatively impacts balance in older individuals (Addison et al., 2014b, 2017) and is found to be higher in fallers than non-fallers (Addison et al., 2014a). Accordingly, the amount of IMAT in the muscle appears to have high clinical relevance. However, to the date, there is only one study (Yoshida et al., 2012) that examined the influence of IMAT on central activation. In the knee extensor muscles, it was previously demonstrated that central activation (measured by central activation ratio, CAR, during an isometric contraction) was inversely associated with IMAT in older adults (Yoshida et al., 2012). The measure of central activation measured by CAR, provides a quantification of muscle activation by using force measurements by superimposing an electrical stimulus during a maximal contraction (Bampouras et al., 2006). However, the use of electrical stimulation to examine muscle activation is not as common or as easily applied as sEMG, which is consistently used in the literature to report neuromuscular activation. Thus, it remains unknown if IMAT influences neuromuscular activation as measured by sEMG. Moreover, a decreased ability to fully activate the muscle may impair an older adult’s ability to recover from a balance perturbation, placing them at an increased risk for falls; thus, further investigation of the influence of IMAT on muscle activation is needed.

The ability to activate the hip abductor muscles is important to recover balance and avoid falls (Mercer et al., 2009; Marques et al., 2013; Lee et al., 2017). For instance, higher gluteus medius (GM) activation, measured by sEMG, is associated with an increased ability to perform a step (Mercer et al., 2009; Marques et al., 2013), which is an essential movement in fall avoidance and balance recovery. Considering sEMG is often used in the assessment of clinical populations (i.e., neurological diseases), and the hip abductor muscles are important in mobility and balance recovery, understanding the influence of IMAT on the sEMG amplitude of the hip abductor muscles may offer insight in the interpretation of sEMG. Therefore, the purpose of this study was to investigate the influence of GM IMAT on sEMG amplitude during a maximal voluntary isometric contraction (MVIC) of the hip abductors in older adults. Because the amount of lean tissue may also influence the sEMG amplitude, a secondary aim was to investigate the influence of GM high-density lean tissue (HDL; muscle free from fat) on sEMG amplitude during MVIC of the hip abductors in older adults. It was hypothesized that the sEMG amplitude would be inversely associated with IMAT and positively associated with HDL in older adults.

## Materials and Methods

### Subjects

Healthy community-dwelling older adults were recruited for this study. All participants provided written informed consent, and the study was approved by the Institutional Review Board of the University of Maryland, Baltimore. As previously described (Inacio et al., 2018), inclusion criteria included healthy, ambulatory older adults with a BMI of less than 35 kg/m^2^. Exclusion criteria included: cognitive impairment (Folstein Mini-Mental Score Exam < 24), sedative use, depression, or a clinically significant functional impairment related to a musculoskeletal, neurological, cardiopulmonary, or metabolic disorder. All inclusion and exclusion criteria were verified by a medical exam with a licensed physician.

### Muscle Composition

**FIGURE 1:**
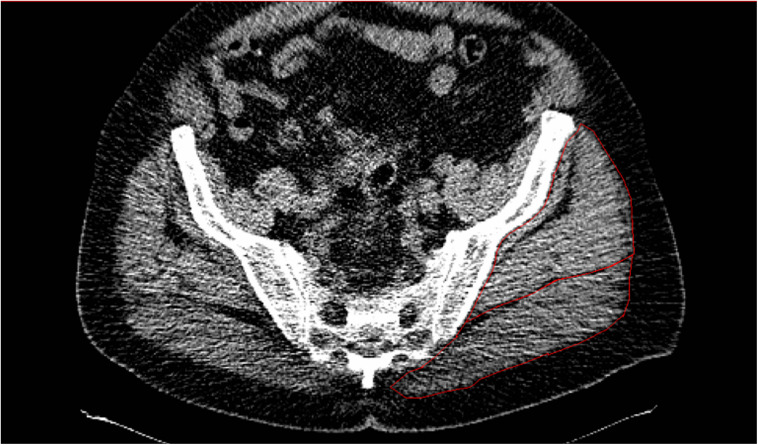
CT scans of one individual demonstrating low attenuation and high levels of IMAT in the gluteal muscles (outlined in red).

Details of the spiral computerised tomography (CT) exam have been published previously (Addison et al., 2017). Briefly, a CT scan was performed starting at L2-L3 and ending at the patella (Siemens Somatom Sensation 64 Scanner). HDL, low density lean tissue (IMAT), and subcutaneous adipose tissue (SUB_FAT_) cross-sectional area (CSA; cm^2^) of the GM and minimus together (Figure 1) were determined using Medical Image Processing, Analysis, and Visualization (MIPAV, v 7.0, NIH) analysis software. For SUB_FAT_ analysis, we verified a strong correlation (*r* = 0.90) between subcutaneous fat thickness and CSA from the CT scans in 50% of the participants; hence, to keep consistency among the study measurements, we used SUB_FAT_ CSA. HDL was defined as 30–100 Hounsfield units (HU), low density lean tissue was representative of IMAT and defined as 0–29 HU and −190 to −30 HU for SUB_fat_. As previously published, LDL and HDL were normalized for the GM muscle size by calculating a percentage of each measure relative to the total muscle CSA (Addison et al., 2017).

### MVIC and sEMG

Participants performed a standing unilateral hip abduction MVIC on a BIODEX System 4 dynamometer (BIODEX, Shirley, NY, United States). The torque signal was recorded at a frequency of 1,500 Hz. The test limb was strapped to the Biodex arm with the thigh pad proximal to the knee joint. Support was provided through the arms by a custom stabilizing frame. Three trials were performed at 30° of hip abduction. Each participant was asked to “push” abduction as fast and hard as possible for ∼5 s with 90 s rest between trials. During the MVIC, sEMG signal was recorded using a wireless sEMG system (NORAXON, United States Inc.) at a frequency of 1,500 Hz. sEMG electrodes arranged in a bipolar configuration (2 cm inter-electrode distance) were placed on the GM in accordance with SENIAM guidelines (Hermens et al., 2000). The sEMG signals were collected in MyoResearch XP software (Noraxon, United States Inc.) and exported to Spike 2 software (CED, Cambridge, United Kingdom) for off-line analysis. Data was filtered (fourth-order Butterworth band-pass filter with a 20–500 Hz pass band), and the root mean square was calculated. The root mean square sEMG amplitude during the MVIC was calculated over a 500 ms epoch, 250 ms either side, from instantaneous maximal voluntary torque (EMG_MVT_) at the highest MVIC.

### Statistical Analysis

A Shapiro–Wilk test was used to assess the normality of the data. Pearson’s product-moment bivariate correlations between sEMG amplitude and each predictor variable (HDL, IMAT, and SUB_FAT_) was performed. Considering the influence of SUB_FAT_ on sEMG (Lanza et al., 2018), partial correlations were performed between sEMG amplitude and muscle composition (IMAT and HDL) with SUB_FAT_ as a covariate. There was no difference in the correlation between absolute (cm^2^) or relative (% CSA) values; hence, all correlations are presented as cm^2^. Additionally, considering possible sex differences, an independent *t*-test was performed for all variables, and no differences was found (*P* ≤ 0.081). Thus, results were presented for both sexes as one group. Statistical analysis was performed using SPSS version 25 (IBM Corporation, Armonk, NY, United States); the significance level was set at *P* < 0.05.

## Results

**FIGURE 2:**
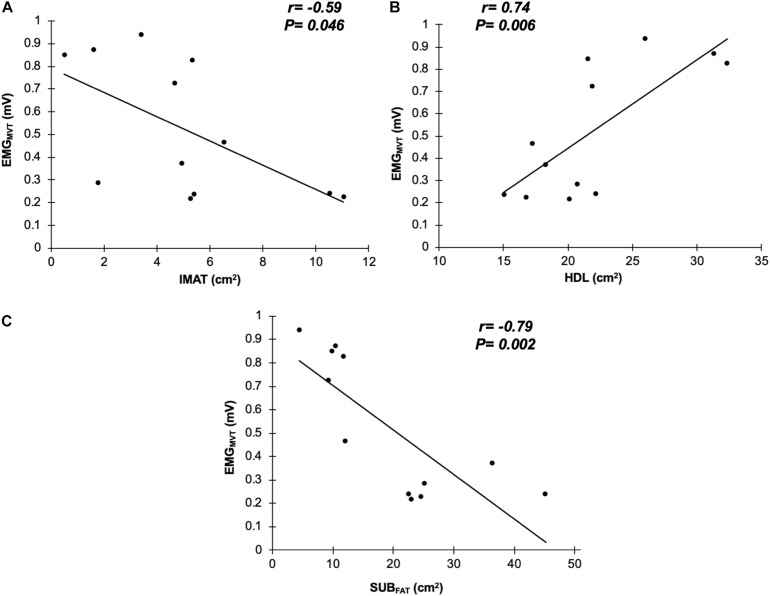
Correlations between surface electromyography at maximal voluntary torque (EMG_MVT_) with intramuscular fat (IMAT; **A**), high density lean tissue (HDL; **B**), and subcutaneous fat (SUB_FAT_; **C**) from gluteus medius and minimus (*n* = 12).

**FIGURE 3:**
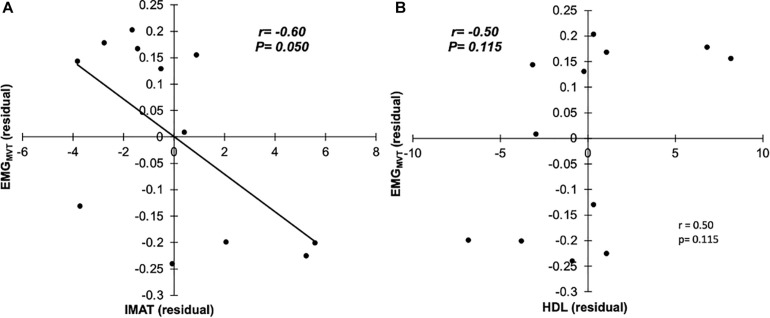
Partial correlations with SUB_FAT_ as covariate, between EMG_MVT_ and IMAT **(A)**, and HDL (**B**; *n* = 12).

Seven males (N = 7; 71.7 ± 3.1 years, 1.73 ± 0.05 m, 83.5 ± 14.1 kg, and BMI: 27.8 ± 4.6 kg/m^2^) and five females (N = 5; 69.6 ± 4.2 years, 1.62 ± 0.05 m, 73.9 ± 8.6 kg, and BMI: 28.3 ± 4.9 kg/m^2^) were tested. There was a significant bivariate negative correlation between GM sEMG amplitude and IMAT (*r* = −0.585, *P* = 0.046, 95%CI: −0.867 to −0.210; Figure 2A) and also SUB_FAT_ (*r* = −0.786, *P* = 0.002, 95%CI: −0.967 to −0.642; Figure 2C). Conversely, there was a significant correlation between GM sEMG amplitude and HDL (*r* = 0.738, *P* = 0.006, 95%CI: 0.490 – 0.903; Figure 2B). When controlling for SUB_FAT_, the partial correlations demonstrated a consistent negative correlation between GM sEMG amplitude and IMAT (*r* = −0.602, *P* = 0.050, 95%CI: −0.936 to −0.077), Figure 3A. However, when accounting for SUB_FAT_, no significant correlation was found between GM sEMG amplitude and HDL (*r* = 0.503, *P* = 0.115, 95%CI: 0.009 – 0.228), Figure 3B.

## Discussion

This study investigated the influence of GM and minimus IMAT on sEMG amplitude during a MVIC of the hip abductors in older adults. We partially confirmed our hypothesis showing that sEMG amplitude was inversely associated with IMAT in older adults, and demonstrated that explained up to 36% of the variance of neuromuscular activation in the hip abductors as measured with sEMG in older adults. To the best of our knowledge, this is the first study to show that neuromuscular activation, measured by sEMG, is influenced by the amount of adipose tissue within the muscle. This relationship was maintained even when controlling for a confounding factor (subcutaneous fat). Thus, we provide evidence that GM neuromuscular activation is potentially altered by the amount of adipose tissue inside the muscle. Moreover, although lean muscle tissue positively influenced sEMG, this association disappeared after accounting for subcutaneous fat. Overall, these results bring important information which may be important in the interpretation of sEMG signals in different populations, especially those of disuse and muscle atrophy who may have increased IMAT.

Previous work demonstrated that the knee extensor neuromuscular activation, measured by CAR, was partially explained by the amount of adipose tissue inside the muscle of older adults (∼26%; Yoshida et al., 2012). While we found similar results, we recognize the differences in techniques and muscle groups may be important. The previous study (Yoshida et al., 2012) did not examine the hip abductors, nor did they measure sEMG; hence, the differences between techniques and muscle groups to measure muscle activation should be further evaluated. When considering the limitations of the CAR technique (Bampouras et al., 2006), an investigation of the sEMG and IMAT of the quadriceps femoris would be essential for clarifying if there are differences between muscle groups. With aging, IMAT increases (Ryan et al., 1996; Goodpaster et al., 2001; Gallagher et al., 2005; Marcus et al., 2010) while lean muscle tissue decreases (Frontera et al., 2000; Hughes et al., 2002; Vandervoort, 2002). Increased total body fat has been associated with impaired neuromuscular activation in sedentary young and older women (Tomlinson et al., 2014), but these previous studies did not examine the relationship with IMAT. Nevertheless, the decrease in lean tissue, which is related to denervation of motor neurons and muscle fibers (Vandervoort, 2002; Piasecki et al., 2018) with a larger reduction in type II fibers (Lexell, 1997), results in a lower capacity to produce force and may also have an impact on neuromuscular activation as seen in our study.

The present study was not designed to explore the mechanism by which IMAT influences neuromuscular activation, but some factors might help explain the present results. Given that, IMAT represents the deposit of lipids within and between muscle fibers (Karampinos et al., 2012), the increase in IMAT may change muscle architecture (e.g., pennation angle and fascicle length). When a contraction is performed, and the sarcomere decreases in width, it is expected a decrease in fascicle length followed by an increase in pennation angle (Ichinose et al., 1997; Rekabizaheh et al., 2016). Thus, the increase in IMAT in older adults may change muscle structure which could impact fascicle length and pennation angle (Rahemi et al., 2015), and ultimately neuromuscular activation (Guilhem et al., 2011; Ghosh et al., 2017). This suggestion may be reinforced by previous studies showing that muscle structure changes with aging, with older adults showing higher amounts of non-muscle tissue (including fat) inside the muscle compared to young adults (Rice et al., 1989; Overend et al., 1992). Future research is warranted to better understand the relationship between increased IMAT and reduced neuromuscular activation.

High density lean partially explained the variance (∼54%) of the neuromuscular activation in older adults. However, after controlling for SUB_FAT_, there was no significant association between variables, albeit did present a moderate correlation (*r* = 0.503). Thus, although HDL did not directly affect muscle activation in the present study, the small number of participants used here may be a limiting factor, and likely, a larger sample size may be required to produce a significant correlation. It could be hypothesized that higher HDL would provide more motor units to be recruited and result in a greater neuromuscular activation. However, it was previously demonstrated that a decrease in lean muscle mass in older adults does not directly translate into a decline in muscle function (Goodpaster et al., 2006; Addison et al., 2014a), which may partially explain the lack of association between HDL and neuromuscular activation seen here. Therefore, the increases in neuromuscular activation in older adults may be more likely to be related to the increased motor unit characteristics (e.g., motor unit recruitment and firing frequency) than lean muscle mass. Nonetheless, this outcome possibly reinforces the fact that increased IMAT appears in some way to directly affect the ability to activate the muscle, as previously demonstrated (Yoshida et al., 2012).

Because the present study had a small sample size, we may have been underpowered to detect some correlations. Note that, conclusions for bivariate correlations should be taken with caution. Nonetheless, it is important to highlight that there are several other extrinsic (e.g., fiber orientation) and intrinsic actors (e.g., fiber diameter) that also influence sEMG amplitude (De Luca, 1997). The images from the CT scan do not allow to properly separate GM from minimus, which may limit our conclusions. Additionally, the older individuals in the present study were healthy, and extrapolation to a non-healthy population should be done carefully. Our results are also limited to the GM muscle, and exploring other muscles would help to clarify our results. Since the present study was not designed to explore the mechanism by which IMAT or HDL influences neuromuscular activation, this is an area for future exploration. For example, future studies should investigate the muscle internal structure (e.g., muscle architecture and/or non-muscle tissue) to further examine the mechanisms by which both IMAT and HDL influence neuromuscular activation. The current results have an important clinical application related to the interpretation of results from sEMG. When interpreting the results in populations that presented augmented fat infiltration (e.g., older adults, those who are obese, or those with neurologic conditions), the influence of IMAT in the ability to activate the muscles should be taking into consideration.

In conclusion, the neuromuscular activation of the hip abductor muscles may be reduced by the quantity of IMAT, while the lean tissue after accounting for subcutaneous fat, may not influence the activation of the GM muscle. Therefore, to better interpret neuromuscular activation from gluteus medius in older adults during a maximal isometric contraction, accounting for the amount of intramuscular fat would provide a more accurate representation of the signal.

## Data Availability Statement

The raw data supporting the conclusions of this article will be made available by the authors, without undue reservation.

## Ethics Statement

The studies involving human participants were reviewed and approved by the Institutional Review Board of the University of Maryland, Baltimore. The patients/participants provided their written informed consent to participate in this study.

## Author Contributions

AR, VG, and OA conceived and designed research. ML and WP perform data processing and analysis. All authors contributed to write, read, and approved the manuscript.

## Conflict of Interest

The authors declare that the research was conducted in the absence of any commercial or financial relationships that could be construed as a potential conflict of interest.

## Abbreviations

CSA: cross-sectional area
CT: computerized tomography
EMG_MVT_: electromyography amplitude from instantaneous maximal voluntary torque
GM: gluteus medius
HDL: high density lean tissue
IMAT: intramuscular fat
MVIC: maximal voluntary isometric contraction
sEMG: surface electromyography.

## References

[B1] Addison O. Inacio M. Bair W.-N. Beamer B. A. Ryan A. S. Rogers M. W. (2017). Role of hip abductor muscle composition and torque in protective stepping for lateral balance recovery in older adults. *Arch. Phys. Med. Rehabil.* 98 1223–1228. 10.1016/j.apmr.2016.10.009 27840133 PMC5425306

[B2] Addison O. Marcus R. L. LaStayo P. C. Ryan A. S. (2014a). Intermuscular fat: a review of the consequences and causes. *Int. J. Endocrinol.* 2014 1–11. 10.1155/2014/309570 24527032 PMC3910392

[B3] Addison O. Young P. Inacio M. Bair W.-N. Prettyman M. G. Beamer B. A. (2014b). Hip but not thigh intramuscular adipose tissue is associated with poor balance and increased temporal gait variability in older adults. *Curr. Aging Sci.* 7 137–143. 24998419 10.2174/1874609807666140706150924 PMC4480674

[B4] Bamman M. M. Newcomer B. R. Larson-Meyer D. E. Weinsier R. L. Hunter G. R. (2000). Evaluation of the strength-size relationship in vivo using various muscle size indices. *Med. Sci. Sport. Exerc.* 32 1307–1313. 10.1097/00005768-200007000-00019 10912898

[B5] Bampouras T. M. Reeves N. D. Baltzopoulos V. Maganaris C. N. (2006). Muscle activation assessment: effects of method, stimulus number, and joint angle. *Muscle Nerve* 34 740–746. 10.1002/mus.20610 17013889

[B6] Biltz N. K. Collins K. H. Shen K. C. Schwartz K. Harris C. A. Meyer G. A. (2020). Infiltration of intramuscular adipose tissue impairs skeletal muscle contraction. *J. Physiol*. 598 2669–2683. 10.1113/JP279595 32358797 PMC8767374

[B7] Correa-de-Araujo R. Addison O. Miljkovic I. Goodpaster B. H. Bergman B. C. Clark R. V. (2020). Myosteatosis in the context of skeletal muscle function deficit: an interdisciplinary workshop at the national institute on aging. *Front. Physiol.* 11:963 10.3389/fphys.2020.00963 PMC7438777 32903666

[B8] De Luca C. J. (1997). The use of surface electromyography in biomechanics. *J. Appl. Biomech.* 13 135–163. 10.1123/jab.13.2.135

[B9] Frontera W. R. Hughes V. A. Fielding R. A. Fiatarone M. A. Evans W. J. Roubenoff R. (2000). Aging of skeletal muscle: a 12-yr longitudinal study. *J. Appl. Physiol.* 88 1321–1326. 10.1152/jappl.2000.88.4.1321 10749826

[B10] Gallagher D. Kuznia P. Heshka S. Albu J. Heymsfield S. B. Goodpaster B. (2005). Adipose tissue in muscle: a novel depot similar in size to visceral adipose tissue. *Am. J. Clin. Nutr.* 81 903–910. 10.1093/ajcn/81.4.903 15817870 PMC1482784

[B11] Ghosh M. D. Kumar D. Arjunan P. S. Siddiqi A. Swaminathan R. (2017). A computational model to investigate the effect of pennation angle on surface electromyogram of Tibialis Anterior. *PLoS One* 12:e0189036. 10.1371/journal.pone.0189036 29216231 PMC5720512

[B12] Goodpaster B. H. Carlson C. L. Visser M. Kelley D. E. Scherzinger A. Harris T. B. (2001). Attenuation of skeletal muscle and strength in the elderly: the Health ABC Study. *J. Appl. Physiol.* 90 2157–2165. 10.1152/jappl.2001.90.6.2157 11356778

[B13] Goodpaster B. H. Park S. W. Harris T. B. Kritchevsky S. B. Nevitt M. Schwartz A. V. (2006). The loss of skeletal muscle strength, mass, and quality in older adults: the health, aging and body composition study. *J. Gerontol. Ser. A Biol. Sci. Med. Sci.* 61 1059–1064. 10.1093/gerona/61.10.1059 17077199

[B14] Guilhem G. Cornu C. Guével A. (2011). Muscle architecture and EMG activity changes during isotonic and isokinetic eccentric exercises. *Eur. J. Appl. Physiol.* 111 2723–2733. 10.1007/s00421-011-1894-1893 21399960

[B15] Hermens H. J. Freriks B. Disselhorst-Klug C. Rau G. (2000). Development of recommendations for SEMG sensors and sensor placement procedures. *J. Electromyogr. Kinesiol.* 10 361–374. 10.1016/S1050-6411(00)00027-24 11018445

[B16] Hogrel J.-Y. (2005). Clinical applications of surface electromyography in neuromuscular disorders. *Neurophysiol. Clin. Neurophysiol.* 35 59–71. 10.1016/j.neucli.2005.03.001 16087069

[B17] Hughes V. A. Frontera W. R. Roubenoff R. Evans W. J. Singh M. A. F. (2002). Longitudinal changes in body composition in older men and women: role of body weight change and physical activity. *Am. J. Clin. Nutr.* 76 473–481. 10.1093/ajcn/76.2.473 12145025

[B18] Ichinose Y. Kawakami Y. Ito M. Fukunaga T. (1997). Estimation of active force-length characteristics of human vastus lateralis muscle. *Cells Tissues Organs* 159 78–83. 10.1159/000147969 9575357

[B19] Inacio M. Creath R. Rogers M. W. (2018). Low-dose hip abductor-adductor power training improves neuromechanical weight-transfer control during lateral balance recovery in older adults. *Clin. Biomech.* 60 127–133. 10.1016/j.clinbiomech.2018.10.018 30343209 PMC6293473

[B20] Karampinos D. C. Baum T. Nardo L. Alizai H. Yu H. Carballido-Gamio J. (2012). Characterization of the regional distribution of skeletal muscle adipose tissue in type 2 diabetes using chemical shift-based water/fat separation. *J. Magn. Reson. Imaging* 35 899–907. 10.1002/jmri.23512 22127958 PMC3292710

[B21] Lanza M. B. Balshaw T. G. Massey G. J. Folland J. P. (2018). Does normalization of voluntary EMG amplitude to MMAX account for the influence of electrode location and adiposity? *Scand. J. Med. Sci. Sports* 28 2558–2566. 10.1111/sms.13270 30030921

[B22] Lee H.-J. Chang W. H. Hwang S. H. Choi B.-O. Ryu G.-H. Kim Y.-H. (2017). Age-related locomotion characteristics in association with balance function in young, middle-aged, and older adults. *J. Aging Phys. Act.* 25 247–253. 10.1123/japa.2015-2325 27705064

[B23] Lexell J. (1997). Evidence for nervous system degeneration with advancing age. *J. Nutr.* 127 1011S–1013S. 10.1093/jn/127.5.1011S 9164286

[B24] Marcus R. L. Addison O. Kidde J. P. Dibble L. E. Lastayo P. C. (2010). Skeletal muscle fat infiltration: impact of age, inactivity, and exercise. *J. Nutr. Health Aging* 14 362–366. 10.1007/s12603-010-0081-82 20424803 PMC3758242

[B25] Marques N. R. Hallal C. Z. Crozara L. F. Morcelli M. H. Karuka A. H. Navega M. T. (2013). Lower limb strength is associated with gait biomechanical abnormalities in older female fallers and non-fallers. *Isokinet. Exerc. Sci.* 21 151–159. 10.3233/IES-130491

[B26] Mercer V. S. Gross M. T. Sharma S. Weeks E. (2009). Comparison of gluteus medius muscle electromyographic activity during forward and lateral step-up exercises in older adults. *Phys. Ther.* 89 1205–1214. 10.2522/ptj.20080229 19778980

[B27] Newman A. B. Kupelian V. Visser M. Simonsick E. M. Goodpaster B. H. Kritchevsky S. B. (2006). Strength, but not muscle mass, is associated with mortality in the health, aging and body composition study cohort. *J. Gerontol. Ser. A Biol. Sci. Med. Sci.* 61 72–77. 10.1093/gerona/61.1.72 16456196

[B28] Overend T. J. Cunningham D. A. Paterson D. H. Lefcoe M. S. (1992). Thigh composition in young and elderly men determined by computed tomography. *Clin. Physiol.* 12 629–640. 10.1111/j.1475-097X.1992.tb00366.x 1424481

[B29] Petrofsky J. (2008). The effect of the subcutaneous fat on the transfer of current through skin and into muscle. *Med. Eng. Phys.* 30 1168–1176. 10.1016/j.medengphy.2008.02.009 18400550

[B30] Piasecki M. Ireland A. Piasecki J. Stashuk D. W. Swiecicka A. Rutter M. K. (2018). Failure to expand the motor unit size to compensate for declining motor unit numbers distinguishes sarcopenic from non-sarcopenic older men. *J. Physiol.* 596 1627–1637. 10.1113/JP275520 29527694 PMC5924831

[B31] Rahemi H. Nigam N. Wakeling J. M. (2015). The effect of intramuscular fat on skeletal muscle mechanics: implications for the elderly and obese. *J. R. Soc. Interface* 12:20150365. 10.1098/rsif.2015.0365 26156300 PMC4535407

[B32] Reid K. F. Doros G. Clark D. J. Patten C. Carabello R. J. Cloutier G. J. (2012). Muscle power failure in mobility-limited older adults: preserved single fiber function despite lower whole muscle size, quality and rate of neuromuscular activation. *Eur. J. Appl. Physiol.* 112 2289–2301. 10.1007/s00421-011-2200-2200 22005960 PMC3394542

[B33] Rekabizaheh M. Rezasoltani A. Lahouti B. Namavarian N. (2016). Pennation angle and fascicle length of human skeletal muscles to predict the strength of an individual muscle using real-time ultrasonography: a review of literature. *J. Clin. Physiother. Res.* 1 42–48. 10.22037/jcpr.v1i2.13712 29708228

[B34] Rice C. L. Cunningham D. A. Paterson D. H. Lefcoe M. S. (1989). Arm and leg composition determined by computed tomography in young and elderly men. *Clin. Physiol.* 9 207–220. 10.1111/j.1475-097X.1989.tb00973.x 2743739

[B35] Ryan A. S. Nicklas B. J. Elahi D. (1996). A cross-sectional study on body composition and energy expenditure in women athletes during aging. *Am. J. Physiol. Metab.* 271 E916–E921. 10.1152/ajpendo.1996.271.5.E916 8944681

[B36] Tomlinson D. J. Erskine R. M. Morse C. I. Winwood K. Onambélé-Pearson G. L. (2014). Combined effects of body composition and ageing on joint torque, muscle activation and co-contraction in sedentary women. *Age* 36:9652 10.1007/s11357-014-9652-9651 PMC4082607 24744050

[B37] Vandervoort A. A. (2002). Aging of the human neuromuscular system. *Muscle Nerve* 25 17–25. 10.1002/mus.1215 11754180

[B38] Yoshida Y. Marcus R. L. Lastayo P. C. (2012). Intramuscular adipose tissue and central activation in older adults. *Muscle Nerve* 46 813–816. 10.1002/mus.23506 23055318

